# The evolving role of structural biology in pharma: integration of X-ray crystallography, cryo-electron microscopy and beyond

**DOI:** 10.1107/S2059798326004390

**Published:** 2026-05-26

**Authors:** Jill E. Chrencik, Hua-Poo Su, Yacob Gomez Llorente, Rachel L. Palte, Daniel J. Klein, Robert P. Hayes, Sadie P. Antine, Guilermo A. Asmar-Rovira, Nicole Bertoletti, Noel J. Byrne, Brittany L. Carroll, Ciro D. Cordeiro, Kaylynn Curfman, James Dutko, Michael J. Eddins, Thierry Fischmann, Barani Govindarajan, Corey W. Hecksel, Thu Ho, Kaspar Hollenstein, Mee Ra Hong, Alan Hruza, Andrii Ishchenko, Maru Jaime-Garza, Matt J. Jaremko, Hayley L. Knox, James Kostas, Harini Krishnamurthy, Ioannis Manolaridis, Cameron L. Noland, Andrea T. Partridge, Sangita B. Patel, Paul Reichert, John C. Reid, Catarina Felisberto-Rodrigues, Chitra A. Shintre, Jennifer M. Shipman, Elakkiya Tamilselvan, Morgan E. Walker, Susanne N. Walker, Renjing Wang, Katharine M. Wright, Tien-Jui Yen, Byung-Kuk Yoo, Sandra B. Gabelli

**Affiliations:** a Discovery Chemistry, MRL, Merck & Co., Inc., South San Francisco, CA94080, USA; b Discovery Chemistry, MRL, Merck & Co., Inc., West Point, PA19486, USA; c Discovery Chemistry, MRL, Merck & Co., Inc., Rahway, NJ07065, USA; d Discovery Chemistry, MRL, Merck & Co., Inc., Boston, MA02115, USA; e Eurofins Lancaster Laboratories, LLC, Lancaster, PA17601, USA; f Evotec (US) Inc., Princeton, NJ08540, USA; g Thermo Fisher Scientific Waltham MA02451 USA; University of Regensburg, Germany

**Keywords:** X-ray crystallography, cryo-EM, cryo-ET, microED, structure-based drug design, drug discovery, drug development, *in situ*, formulations, industrial structural biology

## Abstract

Structural biology has fundamentally influenced pharmaceutical research and development at Merck Sharp & Dohme LLC, Rahway, New Jersey, USA, progressing from pioneering macromolecular crystallography in the 1980s to a fully integrated platform that today encompasses X-ray crystallography, cryo-electron microscopy, micro-electron diffraction and cryo-electron tomography. In this review, we present a comprehensive overview of how these complementary methods have advanced drug discovery across diverse therapeutic areas. We illustrate how atomic to cellular structural insights inform drug discovery and development, from target identification and validation, hit finding, lead identification through lead optimization and clinical progression. Furthermore, we describe how structural biology techniques aid in formulation strategies of antibodies and vaccines. Finally, we highlight the growing integration of *ex situ* and *in situ* approaches as a paradigm shift towards elucidating drug mechanisms in native cellular contexts, a transition poised to accelerate the discovery and development of next-generation therapeutics.

## Abstract 2

This review describes how an integrated structural biology platform at Merck Sharp & Dohme LLC, Rahway, New Jersey, USA, combining X-ray crystallography, cryo-EM, MicroED and cryo-ET, enables seamless three-dimensional insights from molecular to cellular length scales. By uniting *ex situ* and *in situ* approaches with AI/ML-enabled data integration, the platform directly informs target selection, structure-based drug design, formulation strategies and mechanism-of-action studies across multiple therapeutic areas. These advances illustrate a scalable and impactful framework for accelerating mechanism-based, next-generation therapeutic discovery and development.

## 1. Introduction

### 1.1. The techniques that enable us: macromolecular X-ray crystallography, cryo-EM, cryo-ET, MicroED

**Figure 1:**
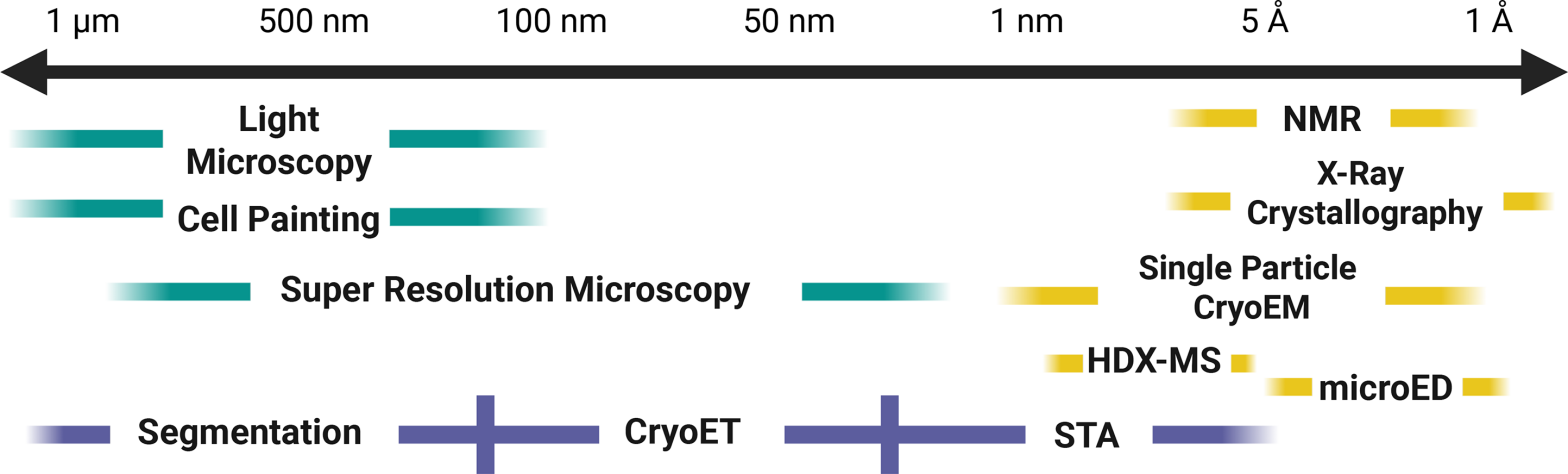
From *ex situ* to *in situ* visualization across a continuum of resolution and scale from 1 Å to ∼1 µm. Structural and cellular biology techniques span complementary spatial scales. Techniques shown on the right (yellow), including NMR spectroscopy, X-ray crystallography, single-particle cryo-EM, hydrogen–deuterium exchange (HDX) and MicroED, represent *ex situ* structural biology approaches, in which purified or reconstituted samples are analyzed outside their native cellular environment. Techniques shown on the left (teal), including light microscopy, cell painting and super-resolution microscopy, represent *in situ* cellular biology methods that enable visualization within intact cells and tissues, preserving biological context. The blue curve highlights cryo-electron tomography (cryo-ET), which uniquely enables *in situ* structural analysis across length scales, from micrometres (∼1 µm) to near-atomic resolution (∼1 Å), through segmentation and correlative workflows at larger scales and subtomogram averaging (STA) at higher resolution. Together, these complementary approaches bridge molecular and cellular length scales, enabling high-resolution structural characterization of drug targets and complexes while also visualizing therapeutic mechanisms and effects within native cellular environments. Created in *BioRender*. Gabelli, S. (2026), https://BioRender.com/kl9m6ul.

Structural biology interrogates the architecture of bio­molecules and the interactions that govern their function, providing the mechanistic insights that underpin modern drug discovery, from identifying and validating targets to designing, optimizing and de-risking candidate therapeutics. Core methodologies, including X-ray crystallography, hydrogen–deuterium exchange (HDX), nuclear magnetic resonance (NMR), cryo-electron microscopy (cryo-EM), micro electron diffraction (MicroED) and cryo-electron tomography (cryo-ET) provide complementary views that collectively span resolution scales from atoms to cells (Fig. 1 ▸). By visualizing targets and their ligands at high resolution, structural biology reveals mechanisms of action, identifies druggable features and guides molecular design tailored to specific conformations and binding sites. Structure-based drug design (SBDD) is inherently iterative, proceeding through repetitive cycles of designing, making, testing and analyzing (DMTA) to refine target affinity, potency, selectivity and overall pharmacological profile while minimizing off-target liabilities. As these techniques are increasingly integrated across discovery and development, structural insights now inform end-to-end decision-making, accelerating the translation of molecular understanding into effective therapeutics.

In practice, X-ray crystallography and cryo-EM are highly complementary (Vénien-Bryan *et al.*, 2017 ▸). Crystallography delivers unparalleled atomic resolution for small to medium-sized proteins and stable complexes, enables fragment-based and structure-guided drug design through precise active-site mapping, and supports robust model refinement with mature validation metrics. Cryo-EM excels at capturing multiple conformational states, visualizing native-like assemblies in lipidic or crowded environments and accommodating modest sample quantities (Renaud *et al.*, 2018 ▸). X-ray crystallography remains the gold standard for achieving iterative atomic-resolution structures and providing precise stereochemical detail. Cryo-EM is particularly powerful for studying large, compositionally heterogeneous complexes that are challenging or impossible to crystallize. Integrated strategies, such as fitting high-resolution crystallographic domains into cryo-EM density maps or using cryo-EM to guide construct design and crystallization, maximize structural insight and offset individual method limitations. Together, these approaches broaden the range of tractable targets and accelerate mechanism elucidation and therapeutic discovery.

Historically, structural biology has relied on methods such as NMR, X-ray crystallography, single-particle cryo-EM, HDX and MicroED (Fig. 1 ▸) that analyze *ex situ* samples, or purified recombinant samples, outside of their native cellular environment and removed from their physiological context. These approaches, which are central to the gene-to-structure strategy of Merck Sharp & Dohme LLC, Rahway, New Jersey, USA (hereafter MSD), demand substantial resourcing, including extensive protein engineering, robust expression and purification pipelines, and access to synchrotron beamlines and state-of-the-art microscopes. Complementing the *ex situ* work, *in situ* techniques such as light microscopy, cell painting and super-resolution microscopy visualize biology at cellular and subcellular scales, framing mechanistic questions. Advances in cryo-ET, coupled with segmentation and sub­tomogram averaging (STA), preserve cellular context, enabling the direct visualization of therapeutic effects in native environments, thus extending *in situ* to the structural biology field. Combining *ex situ* and *in situ* methods create a continuum of resolution, from roughly 1 Å to 1 µm, that connects protein-level mechanisms to organelles and whole-cell architecture. In this framework, structural biology uses *ex situ* approaches to define molecular targets and guide design, while *in situ* imaging extends these insights into living systems, clarifying how cells work and how drugs modulate them. Looking ahead, a major frontier in the field is the integrated visualization of genetic and pharmacological perturbations across diseased and normal tissues. The traditional gene-to-structure paradigm of protein engineering, expression and purification remains essential for producing high-resolution models. Developing capabilities such as *in situ* cryo-ET enable the direct observation of native cellular architecture and drug-induced changes within intact cells and tissues, thereby bridging molecular structures to physiological context.

### 1.2. High-throughput protein expression and purification

**Figure 2:**
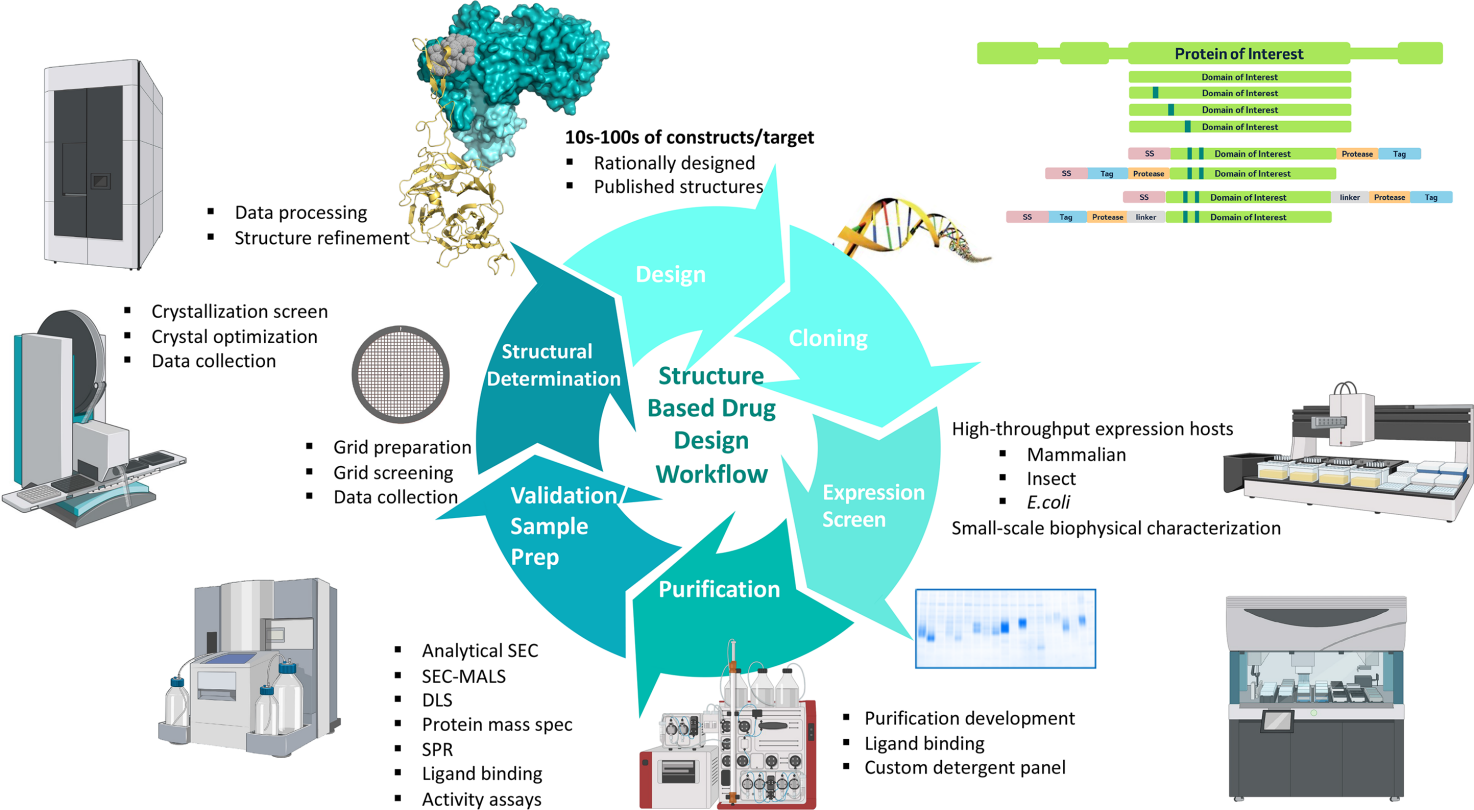
Protein expression and purification DMTA workflow. The schematic illustrates the iterative design–make–test–analyze (DMTA) cycle used to optimize protein constructs for structural biology. Key upstream steps include construct design and cloning, followed by high-throughput expression screening across multiple host systems and purification development using size-exclusion chromatography (SEC), multi-angle light scattering (MALS), dynamic light scattering (DLS), ligand-binding and activity assays. Downstream activities encompass sample preparation for structural studies, crystallization screening, cryo-EM grid preparation, data collection, and computational analysis and refinement. Collectively, these integrated stages enable rapid optimization, rigorous characterization and structural determination of protein targets, supporting mechanistic insight and downstream drug-discovery efforts.

Protein expression and purification for structural biology have evolved from artisanal, low-throughput workflows to highly automated, scalable pipelines capable of evaluating numerous constructs and conditions in parallel. Pioneering consortia such as the Structural Genomic Consortium (SGC), Joint Center for Structural Genomics (JCSG) and North East Structural Genomics Consortium (NESG) helped the field establish standardized, high-throughput cloning, expression screening and multi-step purification workflows using automated liquid handlers, robotic chromatography (FPLC/HPLC) and 96-well formats, dramatically increasing success rates and speed (Elsliger *et al.*, 2010 ▸; Savitsky *et al.*, 2010 ▸; Kraft *et al.*, 2019 ▸; Chandonia & Brenner, 2006 ▸). Today, modular chromatography kits, inline analytics (UV, MALS, MS) and integrated LIMS/ELN systems enable rapid construct triage, parallel purification and reproducible quality control at large scales, while AI-guided construct design and expression optimization further compress the gene-to-structure timeline. Protein expression and purification are foundational to the MSD structural biology pipeline, providing the high-quality protein(s) required for crystallography, cryo-EM and biophysical characterization (Fig. 2 ▸). Standardized workflows, high quality control and efficient allocation of specialized infrastructure (*e.g.* high-throughput expression platforms, chromatography suites and analytical characterization) are essential for the generation of structure-grade protein for structural characterization (Kostas *et al.*, 2024 ▸).

Both macromolecular X-ray crystallography and single-particle cryo-EM (SPA cryo-EM) fundamentally rely on the availability of highly purified, well behaved protein samples, and recent advances in protein-production technologies have strengthened the complementarity between these structural methods. Crystallography traditionally favors exceptionally homogeneous, conformationally uniform specimens capable of packing into ordered lattices, whereas cryo-EM tolerates a greater size and compositional heterogeneity while still delivering near-atomic resolution with less sample for many systems. As a result, the increasing sophistication of upstream expression and purification pipelines now plays a decisive role in expanding the range of biological questions accessible to both techniques.

Methodological innovations in protein expression have reshaped the landscape of structural biology. The choice of host is target-driven: engineered *Escherichia coli* strains for rapid prototyping and optimized for disulfide-bond formation or membrane-protein production, the baculovirus expression vector system (BEVS) for complex or multi-subunit assemblies and HEK/CHO platforms for human post-translational modifications (PTMs) are all now routine, scalable options (Jarvis, 2009 ▸). High-throughput microscale systems such as Ambr^®^ 15 microbioreactor (Sartorius, Germany) and customized liquid handlers such as Tecan Fluent^®^ (Tecan, Switzerland) enable the parallel evaluation of multiple expression conditions with minimal material, reducing time, labor and cost while improving the likelihood of isolating well behaved proteins suitable for downstream structural analysis (Fig. 2 ▸). Additionally, cell-based assays are used to select constructs; for example, flow cytometry is used to identify those with higher plasma-membrane localization of the target protein, indicative of less aggregation and an increased likelihood of proper folding. These innovations reduce the bottlenecks historically associated with large, multidomain and membrane-embedded targets and thereby broaden the pool of candidates suitable for crystallographic or cryo-EM analysis.

Complementing these gains in expression, purification methodologies have evolved to ensure that increasingly complex targets can be recovered in a structurally tractable form. Automated chromatography systems, high-capacity immobilized metal-affinity resins and improved tag-cleavage enzymes support more reproducible workflows and reduce sample loss, a key factor for cryo-EM, where milligram quantities are no longer required but biochemical integrity remains essential. Advances in membrane-protein purification, ranging from amphipols and nanodiscs to novel copolymers, maintain native-like lipid environments that stabilize challenging complexes for both crystallization trials and grid preparation. Structural chaperones such as native binding partners, nanobodies or Fabs are often used to stabilize target proteins by binding defined epitopes and reducing conformational heterogeneity. Incorporation of the chaperones can increase the effective molecular size for cryo-EM analysis and facilitate crystallization by promoting the formation of ordered lattice interfaces. Addition of native binding partners frequently enhances expression and purification yields by improving folding and solubility. Analytical tools, such as aSEC, mass spectrometry, dynamic light scattering (DLS), differential scanning fluorimetry (DSF) *etc.*, enabled rapid assessment of yield, oligomeric state and purity using minimal protein amounts to accelerate timelines from biomass to crystallization or grid preparation. For *in situ* structural studies by cryo-electron tomography, virus-like particles (VLPs) and exosomes can be engineered to present membrane proteins or complex assemblies in native-like contexts, aiding the preservation of conformation and lipid interactions (Gonzalez-Magaldi *et al.*, 2025 ▸). Production typically leverages mammalian expression with co-expression of scaffolding proteins, followed by gentle purification (density gradients, size exclusion) to maintain vesicle integrity for lamella preparation or plunge-freezing.

## 2. Overview

### 2.1. Pioneering industrial structural biology: the MSD Research Laboratories (MRL) perspective

#### 2.1.1. Building industrial-scale macromolecular crystallography

**Figure 3:**
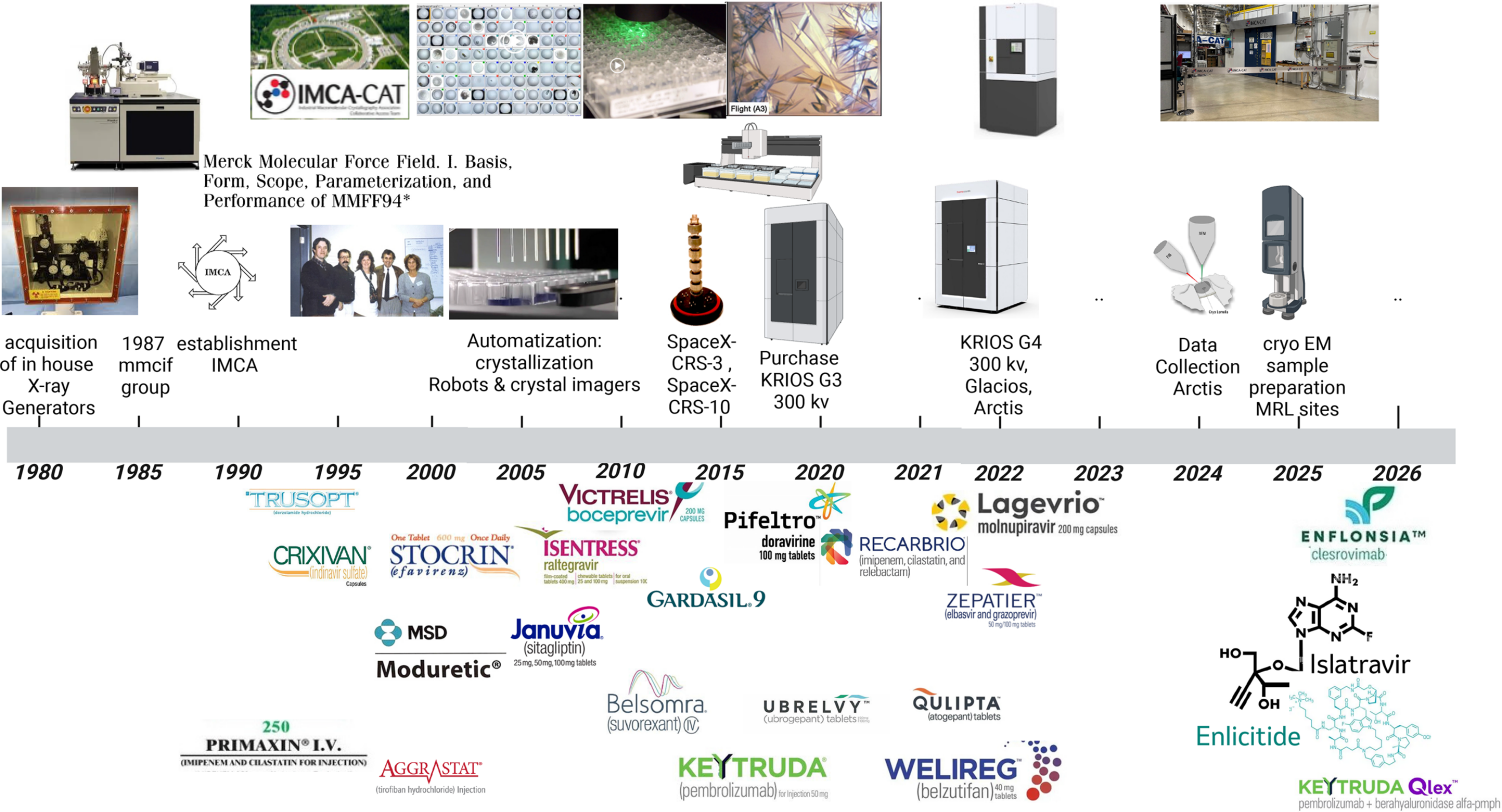
Timeline of the impact of structural biology at MSD (∼1980–2026). Top: evolution of MSD structural biology infrastructure and contributions to the field. Key highlights include early in-house X-ray sources (*e.g.* Nicolet and Rigaku generators); the founding of IMCA; leadership in definition of mmCIF community standards and Halgren’s Merck Molecular Force Field, MMFF94; collaborations in microgravity crystallization on the International Space Station (SpaceX-CRS-3 in 2014 and SpaceX-CRS-10 in 2017 using HH-PCF hardware, with pembrolizumab crystals grown in space); the automatization of crystallography with robots for crystallization, crystallization imagers and UV nanocrystal detectors. Automatization in protein expression and purification is shown with the Ambr 15. 2018 saw the establishment of the Cryo-EM Center of Excellence with the acquisition of a Krios G3 (Thermo Fisher Scientific), with expansion in 2022 to Krios G4, Glacios and Arctis instruments and site-wide availability of Leica and Vitrobot plungers for sample preparation, and subsequent upgrades through 2025. Bottom: representative FDA-approved or late-stage MSD therapeutics enabled or accelerated by structural biology are positioned near their approval/development milestones, illustrating translational impact across the portfolio. Created in *Bio­Render*. Gabelli, S. (2026), https:// BioRender.com/syboeqk.

**Figure 4:**
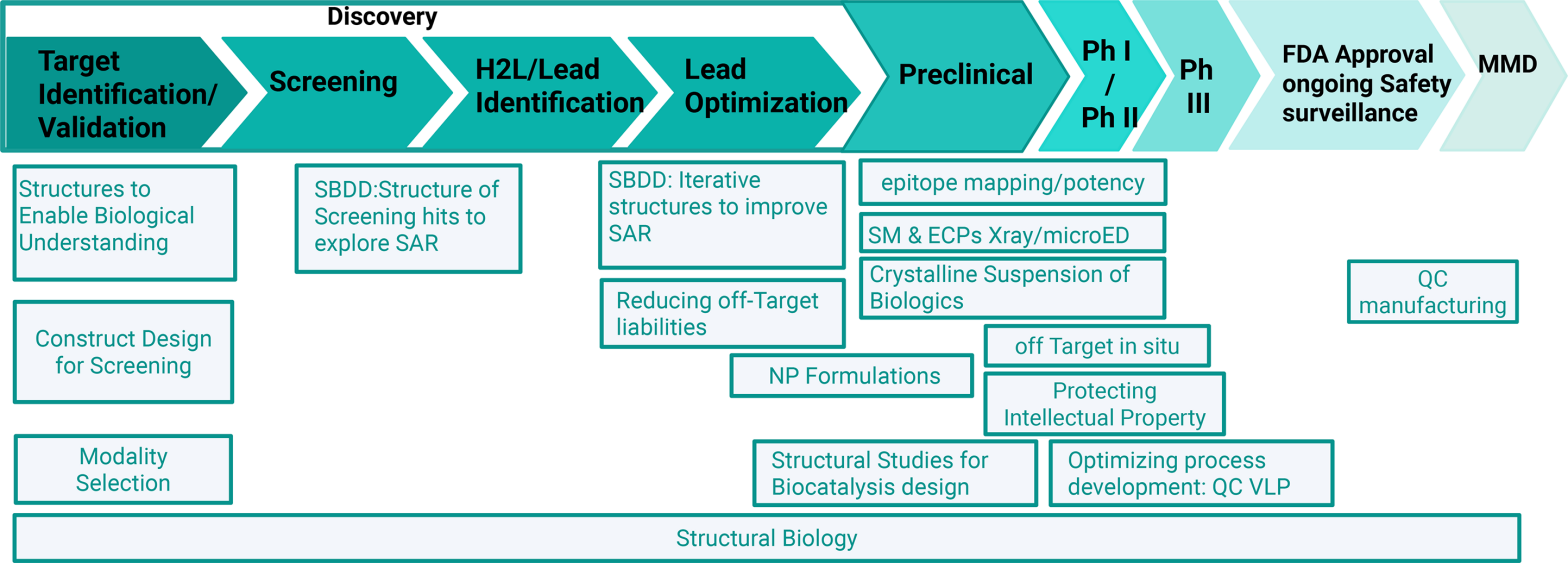
Structural biology across the pharmaceutical R&D continuum, from discovery to development. The schematic illustrates how structural biology supports drug discovery and development activities from target identification through post-approval. The teal arrow traces the discovery-to-development trajectory, including target validation, construct design, screening, hit-to-lead and lead optimization, preclinical testing, clinical development, regulatory approval and post-market safety surveillance. Across this continuum, structural biology enables mechanistic understanding and modality selection, informs construct and assay design and, once tractable chemical matter is identified, guides structure–activity relationships (SAR) and informs structure-based drug design. In late discovery and preclinical development, structural approaches mitigate off-target liabilities (*e.g.* PXR, CYPs, HERG), enable structural studies for biocatalysis engineering and support the generation of crystalline suspensions of biologics. It also allows the structure determination of engineered cyclic peptides and small molecules by X-ray and microED analysis. Downstream applications include antibody epitope mapping and potency assessment, formulation design, optimization of manufacturing processes (*e.g.* quality control of virus-like particles), protecting intellectual property through determination of absolute configuration and *in situ* structural analysis of drug action within tissues. Structural biology continues to inform quality control and product characterization throughout life-cycle management. Created in *BioRender*. Gabelli, S. (2026), https://BioRender.com/ovp1tyq.

Structural biology has influenced pharmaceutical research since the early adoption of macromolecular crystallography. In the 1980s, many pharmaceutical organizations installed in-house X-ray sources – initially systems such as Nicolet^®^ detectors (Thermo Nicolet, USA), followed in the 1990s by instruments such as the Rigaku RU300 rotating-anode X-ray generator (Rigaku, Japan) paired with an R-AXIS IV image-plate detector (Rigaku) (Fig. 3 ▸) – which enabled routine internal data collection and greatly accelerated structure-guided discovery. Building on these capabilities, our organization played a pivotal role in 1992 in establishing the Industrial Macromolecular Crystallography Association (IMCA), an industry beamline consortium which directs the operation of an insertion-device beamline at the Advanced Photon Source (APS) (Industrial Macromolecular Crystallo­graphy Association Collaborative Access Team, 1995 ▸). This high-energy beamline at Argonne National Laboratory was explicitly conceived to meet the demanding needs of structural biology in pharmaceutical drug discovery, enabling rapid structure determination, fragment screening, ligand optimization and the study of challenging targets. Through its role as a founding member, MSD drove a step-change in structural biology capabilities, accelerating discovery and deepening scientific insights. IMCA-CAT (Collaborative Access Team) began delivering X-rays in 1998, providing high-brilliance, tunable synchrotron radiation that dramatically improved data quality, throughput and access to experimental modalities such as multiwavelength and single-wavelength anomalous dispersion. The combination of robust home-source operations and dedicated synchrotron access transformed industrial crystallography from a specialized capability into a scalable engine for medicinal chemistry, materially advancing hit-to-lead and lead-optimization workflows (Fig. 4 ▸). Today, IMCA-CAT operates a state-of-the-art insertion-device (ID) beamline for proprietary pharmaceutical macromolecular crystallography. IMCA-CAT partners include MSD, Abbvie, Bristol Myers Squibb, Evotec, Johnson and Johnson, Novartis, Pfizer and Relay Therapeutics to drive the vision for the beamline (Industrial Macromolecular Crystallography Association Collaborative Access Team, 1995 ▸; Keefe & Stoll, 2019 ▸). The beamline can be tuned from 5 to 20 keV and delivers a flux of 2.0 × 10^13^ photons s^−1^ at the sample. Beam sizes range from 5 to 50 µm supported by compound refractive lenses (CRLs) and an Oxford Instruments Cryojet for cryogenic cooling. High throughput is enabled by automation, with capacity for 672 samples across 42 UniPucks using the ACTOR 2S (Rigaku) robot. Additional features include diffraction rastering with automatic or manual loop centering. Over the last forty years, our organization has shifted from a hybrid model, splitting data collection between in-house instruments and synchrotron sources, to relying entirely on synchrotron facilities worldwide.

#### 2.1.2. Building automation and high throughput in structural biology

MRL acquired and integrated crystallization robotics in the 2000s and 2010s, particularly platforms such as the Mosquito^®^ (TTP Labtech, UK) and Formulatrix systems – Phoenix^®^ HT, NT8, Formulator^®^ and Rock Imager^®^ (Formulatrix, USA) – which enabled nanolitre-scale liquid handling, reducing protein consumption to as little as 50–300 nl per condition, making it feasible to screen hundreds or thousands of crystallization conditions with minimal material (Fig. 3 ▸; Jenkins & Cook, 2004 ▸; Stevens, 2000 ▸). Automated imaging systems such as the Rock Imager that provide temperature-controlled storage and scheduled image acquisition of crystallization experiments eliminate tedious manual drop inspection and enable remote monitoring of crystal growth. Together, these technologies increased throughput from dozens to thousands of experiments per day, providing automated crystal imaging, automated scoring, improved reproducibility by reducing human error and accelerated structure-based drug-design (SBDD) programs.

While X-ray crystallography remains the workhorse for atomic-resolution structure determination, particularly for proteins under 60 kDa, the ‘resolution revolution’ in cryo-EM continues to push boundaries and close the gap with X-ray crystallography. Cryo-EM is revolutionizing structural biology by enabling the visualization of large macromolecular complexes (typically >100 kDa), membrane proteins in native-like lipid environments, dynamic conformational states and heterogeneous samples. Reflecting this shift, MSD acquired its first cryo-EM microscope in 2018: a Titan Krios^TM^ G3i transmission electron microscope (Thermo Fisher Scientific, Waltham, Massachusetts, USA) equipped with a K3^®^ direct electron detector (Gatan, Pleasanton, California, USA) (Fig. 3 ▸). In 2022, the company expanded its capabilities with the acquisition of a Titan Krios^TM^ G4 (Thermo Fisher Scientific), a 300 kV cold field emission gun (cold-FEG) instrument equipped with a Selectris X^TM^ energy filter, Falcon^TM^ 4i direct electron detector and Ceta-D^TM^ CMOS camera (Thermo Fisher Scientific) for high-resolution imaging; a Glacios^TM^ 200 kV cryo-TEM (Thermo Fisher Scientific) equipped with a Selectris X^TM^ energy filter, Falcon^TM^ 4i direct detector and Ceta-D^TM^ CMOS camera for robust routine operation; and an Arctis^TM^ cryo-focused ion beam/scanning electron microscope (cryo-FIB/SEM; Thermo Fisher Scientific) system, enabling studies of both *ex situ* and *in situ* samples (Fig. 3 ▸).

#### 2.1.3. Growing models for SBDD and enabling FAIR data

MRL has been a leader in pioneering efforts for next-generation data standardization. In 1990, the International Union of Crystallography (IUCr) formed a working group to define the macromolecular crystallographic information file (mmCIF; Bourne *et al.*, 1997 ▸; Fitzgerald *et al.*, 2021 ▸). The goal of the mmCIF was to achieve a coherent information flow that allowed the facile retrieval of protein information. mmCIF was introduced as an extension of the CIF format to provide a robust, extensible framework for representing increasingly large and complex macromolecular structures (Fig. 3 ▸). It overcomes the limitations of the legacy PDB format by supporting richer metadata, accommodating complex chemistry and enabling accurate representation of large assemblies and advanced experimental methods. As the official archival standard of the Protein Data Bank, mmCIF ensures that structural data are FAIR (findable, accessible, interoperable and reusable), facilitating reliable data sharing, validation and reuse across the global scientific community. The multi-year project, led by MSD colleague Paula Fitzgerald and which began in 1987 and culminated in 1996, showcased the critical role that MSD colleagues played in the structural biology community (Fig. 3 ▸).

The MRL computational chemistry team developed the MSD Molecular Force Field (MMFF94), a general-purpose, small-molecule potential that delivered reliable geometries, conformational energetics and intermolecular terms across a broad chemical space of drug-like organics, far beyond the narrow functional group coverage of many earlier force fields (Fig. 3 ▸). Still widely used today, MMFF94 was spearheaded by a team of scientists including Thomas A. Halgren, who documented the force field in a five-paper series published in the mid-1990s that covered bond lengths and angles, intermolecular van der Waals, geometries and vibrational frequencies, conformational energetics and extensions to additional chemistries and ions (Halgren, 1996 ▸). The MMFF94 was pivotal for 1990s structural biology and structure-guided discovery, where it underpinned ligand preparation, conformer generation and docking/pose refinement.

#### 2.1.4. Macromolecular crystallization under microgravity conditions

MRL has led foundational research on protein crystallization in microgravity, partnering with NASA and the Center for the Advancement of Science in Space (CASIS) to conduct studies aboard the International Space Station (ISS) since the early 1990s. Leveraging microgravity’s unique environment has informed the development of biologic therapeutics transitioning from intravenous (IV) infusion to subcutaneous (SQ) administration, broadening treatment-delivery options for patients. Early exemplars include insulin and interferon alfa (Intron A), whose drug substances were formulated as crystalline suspensions; these biologics took part in space-shuttle and ISS experiments beginning in 1994 (Long *et al.*, 1994 ▸). Microgravity affords control over three key variables that are difficult to achieve on Earth, elimination of gravity-driven sedimentation, altered diffusion dynamics and enhanced thermal stability, promoting larger, more uniform crystals, reducing turbulence, improving impurity rejection during growth and enabling the precise control of temperature-dependent crystallization processes. Building on this foundation, in partnership with the ISS National Laboratory, we conducted protein crystallization experiments on the SpaceX Commercial Resupply Services-3 mission (SpaceX-CRS-3) in 2014 (Fig. 3 ▸). In 2017, we extended this work to pembrolizumab (Keytruda) on SpaceX-CRS-10. Microgravity, unexpectedly, yielded a uniform crystalline suspension with particle sizes about one-tenth of those observed in parallel ground-based studies, improving viscosity and injectability. Subsequent process development reproduced these microgravity-derived particle attributes on Earth, and the strategy was incorporated into the lead formulation (Reichert *et al.*, 2019 ▸). This microgravity-enabled crystallization of a full-length monoclonal antibody produced a homogeneous crystalline suspension with favorable viscosity and rheological properties.

### 2.2. Pioneering industrial structural biology: from structures to medicines

Drug discovery and development typically span about 10–15 years from target identification to regulatory approval (Fojo, 2023 ▸). Consistent with this timeline, the first commercial therapeutics influenced by structure-based drug design (SBDD) were approved roughly 15 years after the widespread adoption of in-house X-ray crystallography sources (Fig. 3 ▸, bottom). MSD played a foundational role in establishing modern structure-based drug design by demonstrating, for the first time at industrial scale, how high-resolution protein structures could directly inform medicinal chemistry. A representative example is dorzolamide (Trusopt), a carbonic anhydrase inhibitor developed internally and approved by the US FDA in 1994 for the treatment of glaucoma and ocular hypertension (Anderson, 2003 ▸; Rondeau & Schreuder, 2015 ▸; Fig. 3 ▸, bottom). Its design leveraged high-resolution structural insights into human carbonic anhydrase II, including the positioning of His64 and an active-site water molecule upon inhibitor binding (Smith *et al.*, 1994 ▸; PDB entry 1cil). Dorzolamide is widely regarded as the first marketed drug whose design was explicitly guided by atomic-level structural insights, providing a clear proof of concept for SBDD across the pharmaceutical industry. Similarly, SBDD underpinned the development of transformative antiretroviral therapies, including the HIV protease inhibitor indinavir (Crixivan) and the non-nucleoside reverse transcriptase inhibitor efavirenz (Stocrin/Sustiva), reinforcing the power of structural biology to enable rational optimization of potency, selectivity and safety, firmly establishing SBDD as a central pillar of modern drug discovery (Navia *et al.*, 1989 ▸; Chen *et al.*, 1994 ▸; Hartman *et al.*, 1992 ▸).

Over the past four decades, structural biology has contributed to the discovery or optimization of multiple therapeutic agents across multiple disease areas. In infectious diseases, structure-guided efforts enabled agents targeting Gram-negative bacterial infections, imipenem, cilastatin and relebactam (marketed together as Recarbio), as well as antivirals for HCV [boceprevir (Victrelis), elbasvir/grazoprevir (Zepatier)], HIV-1 infection [doravirine/Pifeltro; Côté *et al.*, 2014 ▸) and raltegravir], RSV (clesrovimab/Enflonsia) and COVID-19 (Lagevrio). In oncology, structural insights supported the development of small-molecule and biologic therapies including belzutifan (Welireg) and the immune checkpoint inhibitor pembrolizumab (Keytruda), spanning multiple malignances (Scapin *et al.*, 2015 ▸). In neurological disorders, structure-based design enabled treatments for insomnia (suvorexant/Belsomra) and migraine (ubrogepant/Ubrelvy and atogepant/Qulipta). Contributions to metabolic disorders and cardiovascular diseases include amiloride (Moduretic) for hypertension and edema, tirofiban (Aggrastat), indicated for the reduction of thrombotic cardiovascular events in patients with non-ST-elevation acute coronary syndrome (NSTE-ACS), sitagliptin (Januvia) for type 2 diabetes mellitus, and the oral PCSK9 inhibitor enlicitide, which had reached Phase III CORALreef trials for LDL-cholesterol lowering and cardiovascular risk reduction by 2025 (Navar *et al.*, 2026 ▸; Catapano *et al.*, 2026 ▸; Ballantyne *et al.*, 2026 ▸. Collectively, these examples span enzyme inhibitors, antiretrovirals, checkpoint-targeting antibodies, peptide therapeutics and vaccines, illustrating diverse modes of impact from structural biology (Fig. 3 ▸, bottom). The following sections highlight representative case studies and late-stage programs that underscore this breadth of contribution and illustrate different modes of impact (Fig. 3 ▸, bottom).

### 2.3. Structural biology impacts from drug discovery through development

At MSD, structural biology informs every stage of the drug discovery and development continuum (Fig. 4 ▸). From target identification and validation onwards, structural insights elucidate mechanisms of action and guide protein-construct design. They also play a critical role in modality selection by revealing how the three-dimensional architecture, conformational dynamics and molecular context of a target influence the feasibility, selectivity and developability of different therapeutic approaches, whether small molecules, peptides, antibodies or emerging modalities such as molecular glues or protein degraders. Structural biology further enables efficient execution of the DMTA (design–make–test–analyze) cycle by grounding design hypotheses in atomic-level understanding, guiding molecular-engineering choices, informing experimental prioritization and sharpening structure-based analyses that connect chemical modifications to functional outcomes. Following successful screening campaigns, structural biology supports hit-to-lead (H2L) and lead optimization. As promising chemical matter emerges, teams perform iterative structural analyses of compound–target complexes to interrogate structure–activity relationships (SARs) and connect molecular features to biological outcomes. This workflow is enabled by close, cross-functional collaboration among computational scientists, structural biologists, medicinal chemists and biologists.

Minimizing off-target effects – unintended interactions of chemical matter with nonprimary protein targets – is essential to improving the therapeutic index and reducing adverse events (Fig. 4 ▸). A powerful strategy to mitigate these liabilities is to determine atomic-resolution structures of the off-target protein in complex with the chemical matter of interest. High-resolution techniques such as X-ray crystallography and cryo-EM can reveal the precise binding mode, key molecular contacts and conformational states that drive off-target recognition. These insights enable structure-guided optimization to systematically weaken undesired interactions while preserving or enhancing on-target potency.

Structural biology is increasingly central to decision-making across the discovery-to-development continuum. Within MSD’s vast portfolio, structure-enabled design informs both small- and large-molecule programs through close partnership with medicinal chemistry, pharmacology, therapeutic areas and drug metabolism and pharmacokinetics (DMPK). High-quality structures, paired with clear structure–activity and structure–mechanism narratives, enable faster, more confident progression from hit to lead, and provide the evidentiary basis for go/no-go decisions on chemical matter in priority programs. Beyond early discovery (Fig. 4 ▸), atomic-resolution information during lead optimization guides potency, selectivity and physicochemical tuning while de-risking off-target liabilities. As programs transition into development, structures support translational pharmacology by clarifying cross-species binding modes, informing biomarker strategies and contextualizing resistance mechanisms or variant sensitivity. In chemistry, manufacturing and controls (CMC) and development, structural analyses identify aggregation hotspots and epitope liabilities, which inform developability and address manufacturability constraints for biologics; for small molecules, they inform polymorph risk and enable rational salt and form, including crystallization, to evaluate delivery routes such as subcutaneous administration.

#### 2.3.1. CryoEM epitope mapping

CryoEM epitope mapping pinpoints the precise antigenic sites bound by antibodies on native-like structures, resolving binding orientation, footprint and conformational specificity across different antigen states (Fig. 4 ▸). Electron microscopy polyclonal epitope mapping (EMPEM) extends this to polyclonal sera, deconvoluting the mixture of antibody specificities to quantify immunodominant epitopes and visualize how vaccination or infection shapes the antibody landscape. Furthermore, EMPEM can support the selection of antibodies for potency assays.

#### 2.3.2. Structure determination of macrocyclic peptides

Selecting the optimal solid form of an active pharmaceutical ingredient (API) is critical for scaleup, formulation and clinical development. Structure determination of macrocyclic peptides and small molecules by X-ray crystallography or MicroED provides definitive atomic-level information (protonation state, stoichiometry, hydrogen-bonding networks and packing) that unambiguously distinguishes cocrystals from salts and differentiates true polymorphs from solvates or hydrates (Fig. 4 ▸). This structural clarity strengthens patent claims and freedom to operate by precisely defining the claimed form and its distinguishing features, supporting robust characterization packages (unit cell, space group, diffraction patterns) and reproducible preparation methods. It also enables rigorous assessment of novelty and non-obviousness, evaluation of equivalence or infringement and setting of quality specifications that ensure consistent manufacturing of the protected form. MicroED further expands these capabilities by enabling atomic-level structural determination from nanocrystals that are too small for traditional X-ray crystallography (Newman *et al.*, 2022 ▸). MicroED provides a bridge between cryo-EM and traditional crystallographic methods for small-molecule compounds, particularly when crystals cannot be grown to dimensions suitable for X-ray diffraction or when sample quantity is limited, as is common in early-stage drug development (Fig. 3 ▸; Danelius *et al.*, 2023 ▸). Typically, MicroED crystals measure in the range of 200–300 nm, about nine orders of magnitude smaller than a typical 100 µm crystal used in X-ray crystallography (Danelius *et al.*, 2023 ▸; Nicolas *et al.*, 2025 ▸).

#### 2.3.3. Crystalline suspensions enable subcutaneous delivery of high-concentration biologics

Converting a therapeutic protein into a highly concentrated crystalline suspension with controlled particle size and stability reduces solution viscosity, improves syringeability and allows higher doses to be delivered in small volumes (Zheng *et al.*, 2026 ▸). Crystallization also enhances physical stability by limiting aggregation and protecting the protein from degradation pathways common in high-concentration liquid formulations. Because particle size and crystal morphology can be precisely tuned through formulation parameters, crystalline suspensions are compatible with autoinjectors and other subcutaneous delivery devices while supporting robust manufacturability and long-term storage.

#### 2.3.4. Nanoparticle (NP) formulations

CryoEM characterization of nanoparticles such as lipid nanoparticles (LNPs), RNA nanoparticles (RNP) and virus-like particles (VLPs) is accelerating pharmaceutical R&D and manufacturing by providing direct, high-resolution structural readouts that guide formulation, process development and quality control (Fig. 4 ▸). For LNPs, cryo-EM enables accurate measurement of particle size and size distribution, as well as assessment of lamellarity and cargo-encapsulation efficiency, thereby supporting formulation development decisions and strengthening quality control (Zhao *et al.*, 2014 ▸). For VLPs, single-particle reconstructions can resolve capsid symmetry, particle integrity, antigen density and orientation, and sample heterogeneity in both particle size and shape, enabling rational immunogen design and tighter control of assembly conditions. Across types of NP and development stages, cryo-EM enhances quality control and batch-to-batch comparability by providing orthogonal confirmation of size, polydispersity and morphology alongside DLS, nanoparticle tracking analysis and SEC-MALS and by detecting low-abundance subpopulations. It also visualizes aggregation, fusion and structural defects linked to stress, shipping or storage conditions, supports identity and purity testing through class averages and 3D reconstructions characteristic of a given product, and helps to establish structure–function correlations (for example, morphology versus transfection efficiency or immunogenicity) that enable the definition of actionable specifications.

In the design, development and engineering of biocatalysts, high-resolution structures of enzymes and enzyme–substrate complexes are transforming sequence- and activity-based screening into hypothesis-driven engineering: active-site and access-tunnel geometries, catalytic residue networks and conformational states resolved by X-ray and cryo-EM guide targeted mutations that enhance activity, selectivity (chemo-, regio-, enantio-) and stability under process conditions (Prier *et al.*, 2019 ▸; Chun *et al.*, 2025 ▸).

Structural biology is expected to see growing engagement with *in situ* interrogation, emphasizing visualization of drug effects within living cells and tissues. Increasing use of cryo-ET *in situ* approaches will enable our ability to observe off-target effects, as well as the optimization of the expression of VLP for development (Fig. 4 ▸).

### 2.4. Building on the PDB legacy: how AI/ML and systematic data curation are reshaping structural biology in pharma

Artificial intelligence (AI) and machine learning (ML) are transforming structural biology workflows in pharmaceutical research, driving efficiencies across the entire drug discovery and development pipeline (Zhang *et al.*, 2025 ▸). It is imperative to recognize that the foundation for the AI/ML revolution in structural biology was laid by the decades-long commitment of the structure community to open data sharing via the Protein Data Bank (PDB), a community-driven resource that now contains over 220 000 structures and has served as the critical training dataset for breakthrough protein structure-prediction models such as *AlphaFold* and *RoseTTAFold* (Berman *et al.*, 2000 ▸; Jumper *et al.*, 2021 ▸; Humphreys *et al.*, 2021 ▸), exemplifying how systematic data recording and curation enables transformative AI applications. The near-experimental-accuracy structure predictions of *AlphaFold*2 were made possible by decades of sustained deposition of crystallographic and NMR structures by structural biologists since 1971 (see, for example, the PDB’s foundational description: Berman *et al.*, 2000 ▸). With the emergence of cryo-EM, the community has sustained this ethos by depositing atomic models in the PDB and associated volumetric maps in the Electron Microscopy Data Bank (EMDB), further enriching the datasets that underpin both traditional and AI-driven structural analyses (Consortium *et al.*, 2023 ▸; Kleywegt *et al.*, 2024 ▸).

In structural biology workflows, next-generation prediction models enable rapid insight prior to experimental structure determination. *AlphaFold*2 (DeepMind), and co-folding algorithms such as *Boltz* and *Chai*, predict three-dimensional protein structures and assemblies, including complexes with DNA, RNA, ligands, ions and post-translational modifications, with near-experimental accuracy, informing construct design and prioritization (Baek *et al.*, 2021 ▸; Passaro *et al.*, 2025 ▸). For multi-chain assemblies and protein–nucleic acid complexes, *RoseTTAFold* and *RoseTTAFoldNA* provide accurate models using unified neural network architectures (Baek *et al.*, 2021 ▸; Humphreys *et al.*, 2021 ▸). *Boltz*-2 (MIT/Recursion) extends co-folding to affinity estimation, offering binding-energy predictions at speeds orders of magnitude faster than traditional free-energy calculations, bridging AI/ML implementations to other areas of drug discovery (Discovery *et al.*, 2024 ▸; Passaro *et al.*, 2025 ▸). Collectively, these tools help structural biologists make informed decisions on construct boundaries, mutation sites for added stability or enhancement of expression, and crystallization strategies before committing to resource-intensive experimental campaigns. Additionally, AI/ML applications now extend upstream into protein expression, purification and crystallization, historically among the most variable and resource-intensive phases of structural biology. By learning from large corpora of experimental outcomes, these tools convert what were once brute-force searches into guided, data-driven optimization further increasing the likelihood of obtaining diffraction-quality crystals or high-resolution cryo-EM particles. Two illustrative examples are *DeepCrystal* and *MARCO* (*MAchine Recognition of Crystallization Outcomes*; Bruno *et al.*, 2018 ▸; Holleman *et al.*, 2021 ▸). The former leverages machine-learning models trained on historical Protein Data Bank (PDB) deposition metadata and crystallization-trial outcomes to prioritize promising conditions. In practice, these systems reduce the experimental search space from thousands of possible buffers, precipitants, salts, pH values and additives to a tractable set of approximately dozens, thereby conserving precious protein material and technician time while improving hit rates (Elbasir *et al.*, 2019 ▸). The latter is a computer vision-based algorithm trained on hundreds of scored protein crystallization images that can be used to score newly acquired crystallization images. In development, ML-driven algorithms assist in refining conformational ensembles and predicting stability under physiological conditions, which informs formulation and therapeutic design. However, despite these advances, experimental validation remains essential to confirm predicted structures and dynamic behaviors, as computational models can be limited by training-data biases and incomplete representation of biological complexity.

Modern cryo-EM SPA workflows increasingly leverage machine learning (ML) through APIs integrated into widely adopted software packages, substantially accelerating data processing while improving reproducibility and map quality. *CryoSPARC*, for example, incorporates ML-driven modules for automated particle picking and rapid 2D/3D classification, enabling high-throughput, unsupervised structure determination with minimal manual intervention (Punjani *et al.*, 2017 ▸). Complementary tools such as *Topaz* apply positive-unlabeled learning to identify particles with limited curated labels, reducing annotation burden and improving recall on challenging datasets (Bepler *et al.*, 2020 ▸). Likewise, *crYOLO* employs deep convolutional networks to deliver fast, accurate and fully automated particle selection across diverse specimen types and imaging conditions (Wagner & Raunser, 2020 ▸). Beyond particle picking, current pipelines are beginning to integrate ML for density interpretation and map refinement, including generative adversarial networks and emerging transformer-based approaches to enhance effective resolution, suppress noise and improve the fidelity of reconstructed maps to enhance effective resolution, suppress noise and improve the fidelity of reconstructed maps (He *et al.*, 2023 ▸; Zhong *et al.*, 2021 ▸).

The full potential of AI/ML in structural biology depends on maintaining a culture of systematic data stewardship. If we treat each protein expression and purification, every crystallization attempt, all X-ray and cryo-EM datasets, and every binding assay as future training data, analogous to how the PDB underpinned *AlphaFold*, we can accelerate the next generation of models. Of particular note is the data recording of negative results and failed experiments, which are equally valuable for ML training. Most organizations are now investing heavily in data infrastructure and standardization protocols to build the high-quality datasets necessary for next-generation ML models (MAINFRAME, Structural Genomics Consortium; https://web-app-153945772792.northamerica-northeast2.run.app; AstraZeneca & BenevolentAI, 2019 ▸). An exemplar of this strategic investment is the MSD–Variational AI collaboration, which targets earlier stages of discovery to improve the efficiency, speed and quality of therapeutic candidates (Pharmaphorum, 2025 ▸). Building on the foundation established by the PDB, sustained investment in experimental data generation today is essential for training predictive models that will further compress drug-discovery timelines tomorrow. Achieving a deep understanding of biology requires an integrated approach that combines advanced computational methods with rigorous experimental work. Together, these complementary strategies pave the way toward revealing the molecular mechanisms of life with increasing precision and insight. Continuous production of high-quality experimental datasets, such as protein expression and purification, X-ray and cryo-EM maps, NMR spectra and kinetic measurements, will improve model accuracy and expand their applicability across targets and modalities (Zhang *et al.*, 2025 ▸). Equally important is robust data capture, standardized metadata and integration into shared repositories, which enable iterative learning and allow AI/ML systems to co-evolve with experimental practice, ultimately yielding more reliable and comprehensive structural biology workflows.

## 3. The impact of structural biology across therapeutic areas

### 3.1. Accelerating the search for broad-spectrum antibiotics

β-Lactamases are a diverse class of bacterial enzymes that hydrolyze the β-lactam ring, inactivating β-lactam antibiotics and rendering them ineffective. The continued rise of β-lactamase-producing strains has driven the development of new antibiotics and β-lactamase inhibitors to counteract this resistance mechanism (Toney *et al.*, 1998 ▸). Our company has been at the forefront of discovering, developing and advancing treatments for serious infections caused by resistant bacteria, contributing agents such as imipenem (discovered by our scientists in the 1980s), the imipenem/cilastatin combination (Primaxin) and the β-lactamase inhibitor relebactam (Figs. 4 ▸ and 5 ▸*a*). Central to these efforts has been elucidating the three-dimensional structures of diverse β-lactamases, which has revealed active-site architectures and catalytic mechanisms of antibiotic inactivation. These structural insights have been pivotal in guiding the design of new inhibitors, including relebactam, and continue to inform rational drug design, enabling more effective strategies against bacterial resistance and improving treatment options for infections caused by resistant pathogens (Blizzard *et al.*, 2014 ▸).

### 3.2. Pioneering research to help address the HIV epidemic

**Figure 5:**
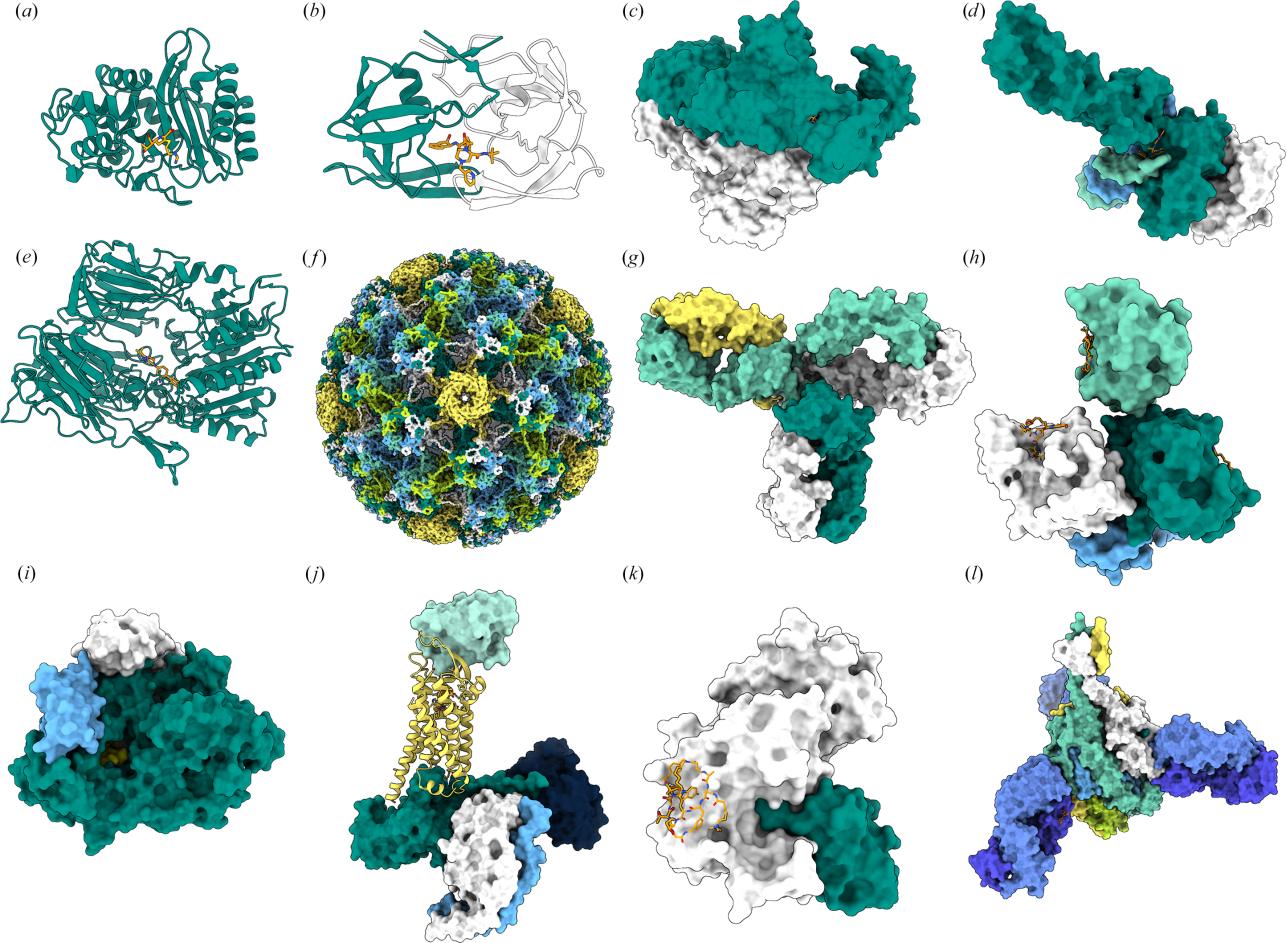
Structural biology impact across selected MSD therapeutic targets. Surface and ribbon representations of molecular targets from selected MSD products whose discovery or optimization was informed by structural biology. Distinct subunits or functional domains are colored to highlight relative positions, interfaces and ligand-binding sites. (*a*) β-Lactamase shown as ribbons (teal) in complex with imipenem (orange; PDB entry 1bt5; Maveyraud *et al.*, 1998 ▸). (*b*) HIV aspartyl protease shown as ribbons (teal and white) with indinavir (Crixivan, orange) depicted in stick representation (PDB entry 1hsh; Chen *et al.*, 1994 ▸). (*c*) HIV reverse transcriptase (PDB entry 3drp; Tucker *et al.*, 2008 ▸). (*d*) HIV integrase (PDB entry 3l2v; Hare *et al.*, 2010 ▸). (*e*) Dipeptidyl peptidase-4 (DPP-4; PDB entry 2p8s; Biftu *et al.*, 2007 ▸). (*f*) Human papillomavirus (HPV) capsid protein fragment (PDB entry 3j6r; Cardone *et al.*, 2014 ▸). (*g*) Pembrolizumab in complex with PD-1 (PDB entry 5dk3; Scapin *et al.*, 2015 ▸). (*h*) Hepatitis C virus (HCV) protein target (PDB entry 3sud; Romano *et al.*, 2012 ▸). (*i*) SARS-CoV-2 RNA-dependent RNA polymerase (PDB entry 7ozu; Kabinger *et al.*, 2021 ▸). (*j*) Orexin receptor (active state; PDB entry 7l1u; Hong *et al.*, 2021 ▸). (*k*) PCSK9 (PDB entry 3h42; Chan *et al.*, 2009 ▸). (*l*) Respiratory syncytial virus (RSV) fusion (F) protein (PDB entry 6ous; Tang *et al.*, 2019 ▸). Created in *BioRender*. Gabelli, S. (2026), https://BioRender.com/hbesh6q.

In 1986, MSD initiated its HIV research program with a dedicated laboratory established at its West Point site, driven by the urgent need to find effective treatments for the global HIV/AIDS crisis. By 1989, our scientists made a landmark breakthrough, using X-ray crystallography to determine the three-dimensional structure of the HIV-1 aspartic protease enzyme: the first HIV protein ever to have its structure unveiled (Navia *et al.*, 1989 ▸). This achievement provided an unprecedented window into the molecular workings of the virus, allowing researchers to decipher the enzyme’s mechanism and uncover the structural cues that could lead to drug resistance. Armed with these insights, our scientists pioneered the development of protease inhibitors, culminating in the discovery of Crixivan (indinavir), a milestone in antiretroviral therapy (Fig. 5 ▸*b*; Chen *et al.*, 1994 ▸).

Building on this foundation, our researchers expanded their focus to other critical HIV enzymes, reverse transcriptase (RT) and integrase, both essential for the replication of the virus and its integration into the host genome (Figs. 5 ▸*c* and 5 ▸*d*). Advanced structural biology techniques, including X-ray crystallography and, more recently, cryo-electron microscopy (cryo-EM), have played a pivotal role in mapping the intricate architecture of these viral proteins. High-resolution structures of reverse transcriptase and integrase provided by X-ray and cryo-EM have revealed the exact binding sites for inhibitors, explained how mutations confer resistance and guided the rational design of next-generation therapies. Specifically, SBDD guided the design of doravirine, a next-generation non-nucleoside reverse transcriptase inhibitor (NNRTI) which binds to an allosteric site on the HIV-1 RT and induces a conformational change that disrupts the ability of the enzyme to convert viral RNA into DNA, thereby blocking viral replication (Côté *et al.*, 2014 ▸).

A key innovation has been the development of islatravir (Figs. 3 ▸ and 5 ▸*c*), a nucleoside reverse transcriptase translocation inhibitor, where the dynamic structure and mechanism of RT has been elucidated, guided by structural biology. Structural investigation of HIV RT with DNA and 4′-ethynyl-2-fluoro-2′-deoxyadenosine (EFdA, MK-8591, Islatravir) in pre- and post-translocation states provided a detailed mechanistic understanding of the exceptional potency, the unique mechanism of action and the high barrier protecting against viral resistance for this important nucleoside reverse transcriptase translocation inhibitor. More recently, the discovery of MK-8527 provides an NRTTI that has potential for extended duration dosing for prophylaxis (Raheem *et al.*, 2025 ▸).

Today, our commitment continues: we leverage these powerful structural insights to develop targeted, effective HIV treatments and prevention strategies, reaffirming our legacy of innovation in combating HIV/AIDS.

### 3.3. Informing the treatment of diabetes

Our company has helped advance diabetes treatment through the development of therapies such as Januvia (sitagliptin). Sitagliptin is a selective inhibitor of dipeptidyl peptidase-4 (DPP-4), the enzyme that rapidly degrades the incretin hormones GLP1 and GIP (Fig. 3 ▸). By inhibiting DPP4, sitagliptin increases circulating incretin levels, which enhances glucose-dependent insulin secretion and helps improve glycemic control in adults with type 2 diabetes. Structural studies, including X-ray crystallography of DPP4 and structure-guided medicinal chemistry, clarified key molecular interactions in the active site of the enzyme and enabled the design and optimization of sitagliptin and related analogs as potent, selective inhibitors. High-resolution X-ray crystal structures of DPP4 revealed a deep catalytic pocket adjacent to a distinctive hydrophobic ‘S1’ subsite and an extended groove (often termed S2/S2 extensive) that accommodates inhibitors (Fig. 5 ▸*e*). These structures mapped the positions of the catalytic serine and nearby residues that form key hydrogen-bond and π-stacking interactions, as well as ordered water molecules that mediate binding. These structural insights guided precise modifications that increased potency into the low-nanomolar range, sharpened selectivity for DPP4 and improved oral bioavailability and metabolic stability (*e.g.* minimizing CYP interactions and avoiding reactive functionalities). Serial cocrystal structures confirmed binding modes and allowed rapid, rational pruning of less promising chemotypes, accelerating the design–make–test cycle. Collectively, this structure-guided approach enabled efficient progression from early leads to a clinically viable, once-daily oral agent, culminating in the approval of Januvia in 2006 (Biftu *et al.*, 2007 ▸).

Structural biology has also illuminated insulin signaling. Using cryoEM, structures were determined of the insulin receptor ectodomain in both unbound and insulin-bound states, revealing the conformational changes that accompany hormone engagement and receptor activation (Scapin *et al.*, 2018 ▸). The insulin receptor is a disulfide-linked dimer composed of two αβ protomers, with the extracellular α subunits forming a complex ectodomain organized into L1, CR, L2 and three fibronectin-type III (FnIII1/2/3) domains. In the unliganded state, the receptor adopts an autoinhibited, ‘inverted V’/‘Λ-shaped’ configuration that limits access to the primary insulin-binding surfaces. Insulin binding induces a transition toward a more compact, ‘T-shaped’ arrangement that brings the membrane-proximal FnIII domains into closer apposition, a prerequisite for transmembrane transmission of the activation signal (PDB entries 6ce9 and 6sof; Scapin *et al.*, 2018 ▸). By mapping these interfaces and conformational transitions, cryo-EM clarified how extracellular ligand binding couples to intracellular tyrosine kinase activation, initiating downstream signaling cascades (IRS recruitment, PI3KAkt pathway and GLUT4 translocation) that regulate glucose uptake and metabolism. The structural framework also rationalizes the effects of naturally occurring and engineered mutations: alterations in the L1–αCT contact surface, FnIII domain interfaces or disulfide linkages can reduce insulin affinity, disrupt conformational coupling or impair dimer geometry, leading to attenuated signaling and clinical phenotypes of insulin resistance or diabetes. These insights guide the interpretation of pathogenic variants, enable structure-informed antibody and ligand engineering, and provide a blueprint for designing receptor modulators that fine-tune insulin signaling.

### 3.4. Contributions to vaccine production and the prevention of HPV

Cryo-EM has made significant contributions to the field of vaccine development, particularly in the production of Gardasil, the human papillomavirus (HPV) vaccine (Figs. 3 ▸ and 5 ▸*f*). Beyond its applications in structural-based drug design, cryo-EM allowed researchers to gain valuable insights into the morphology and assembly of VLPs, that are pivotal for immunogenicity, to optimize production stages. Using cryo-electron tomography, by visualizing the VLPs during the vaccine-production process, scientists can assess their size, shape and structural integrity, ensuring that they mimic the natural HPV structure closely. This detailed morphological characterization is critical, as it helps optimize the formulation process and ensure the efficacy and safety of the vaccine.

Ultimately, the application of cryo-EM in the study of VLPs exemplifies its transformative role in advancing vaccine technology and our work to prevent infectious disease. Additionally, structural biologists have used cryo-EM to conduct epitope-mapping studies which allow identification of the specific regions of the virus that analytical antibodies target (Larpent *et al.*, 2024 ▸). This information is crucial for linking potency assays to the mechanism of action of the vaccine, correlating antibody binding to the relevant epitopes with vaccine efficacy.

### 3.5. Targeting the PD-1 checkpoint inhibitor to treat cancer

Pembrolizumab is a monoclonal antibody that targets the programmed cell death protein 1 (PD-1) receptor, playing a critical role in enhancing the ability of the immune system to fight cancer. Structurally, pembrolizumab is a humanized IgG4κ antibody, which enables it to bind specifically to the PD-1 receptor on T cells, thereby blocking its interaction with ligands PD-L1 and PD-L2. This action reactivates T-cell proliferation and activity against tumor cells, thus promoting antitumor immune responses.

The structure of the human full-length IgG4 S228P anti-PD1 antibody pembrolizumab was determined to a resolution of 2.3 Å by X-ray crystallography (PDB entries 5dk3 and 8sjk; Fig. 5 ▸*g*; Scapin *et al.*, 2015 ▸). Building on the structural insights gained, various innovative formulations of pembrolizumab have been developed with the aim of extending the delivery of pembrolizumab through subcutaneous (subQ) administration as an alternative to the standard intravenous (IV) infusion (Erfani *et al.*, 2023 ▸; Larpent *et al.*, 2024 ▸). Research has focused on optimizing formulation strategies, such as the use of innovative delivery devices and stabilizing excipients, to enhance the pharmacokinetics and bioavailability of the antibody via subcutaneous routes. These advancements aim to improve patient convenience, reduce treatment times and maintain the therapeutic efficacy of pembrolizumab.

### 3.6. Hepatitis C: targeting the protease of hepatitis C virus (HCV)

Hepatitis C virus (HCV) is the major cause of chronic liver disease, leading to cirrhosis and hepatocellular carcinoma. Structural biology has played a crucial role in the development of inhibitors targeting the hepatitis C virus (HCV) nonstructural protein 3 (NS3; Fig. 5 ▸*h*). Detailed structural information, such as the arrangement of the catalytic triad (His57, Asp81, Ser139) and the zinc-binding site, allowed scientists to design specific inhibitors that block enzyme activity. Knowing the structure of the substrate-binding pocket, particularly the S1 pocket, which is shallow and nonpolar and accommodates small hydrophobic residues such as Cys or Thr at the P1 position, was key to the design of selective inhibitors by our company. The structures also revealed the important role of the NS4A cofactor in activating the protease, providing insights into the mechanism of activation and potential allo­steric drug targets. The shallow substrate-binding groove of the NS3 protease of NS3–NS4 is an example of the success that SBDD allows in designing effective small-molecule inhibitors, culminating in the development of successful anti-HCV therapies (Venkatraman *et al.*, 2006 ▸; Prongay *et al.*, 2007 ▸; Parsy *et al.*, 2015 ▸). Drug resistance emerging against first-generation inhibitors further highlighted the need for structural knowledge to design inhibitors targeting new sites or with improved resistance profiles.

### 3.7. Fighting to stop the pandemic: RNA polymerase

The COVID-19 pandemic created a unique opportunity to design and develop oral antiviral agents aimed at halting the progression of mild to moderate cases of the disease. The primary target of interest was the virally encoded RNA-dependent RNA polymerase (RdRP) due to its requirement for viral replication and high level of sequence conservation across variants (Fig. 5 ▸*i*). Molnupiravir, a prodrug of the ribonucleoside analog β-d-*N*^4^-hydroxycytidine (NHC), can be converted in the plasma into NHC and subsequently into its active form, the 5′-triphosphate, through the action of host kinases. Computational studies illustrated that NHC could tautomerize in a fashion that could drive promiscuous base pairing. Subsequently, cryoEM studies of RdRP captured structural snapshots of NHC forming promiscuous base pairs during incorporation and replication, ultimately leading to viral genome mutation and loss of viral fitness (Kabinger *et al.*, 2021 ▸). These structural and computational insights have been leveraged to inform the design of other nucleoside analogs for the treatment of SARS-CoV-2 and other emerging RNA viruses of clinical importance.

### 3.8. Delineating molecular mechanism of action: cryoEM Structures of the active-state orexin receptor

G protein-coupled receptors (GPCRs) are notoriously difficult to characterize structurally due to their pronounced conformational heterogeneity, dynamic coupling to signaling partners, limited stability outside native membranes and often small, flexible ligands that complicate state-specific capture. To overcome these hurdles, we employed a conformation-specific nanobody as a structural chaperone to stabilize the active ensemble and enable cryo-EM determination of orexin receptor 2 (OX2R) in complex with both an endogenous peptide agonist and a small-molecule agonist (Hong *et al.*, 2021 ▸). The extended C-terminal segment of the peptide penetrates the receptor core to reinforce the active conformation, whereas the small molecule binds deep within the orthosteric pocket, engaging analogous key interactions (Fig. 5 ▸*j*). Comparison with antagonist-bound OX2R structures illuminates features that discriminate activation from inhibition and clarifies the state transitions of the receptor. These insights provide a foundation for structure-guided discovery of OX2R agonists, a strategy motivated by the limitations of symptom-focused therapies for narcolepsy type 1 (NT1) and the potential to address the disorder’s underlying orexin pathway deficit. Collectively, these structures have provided a robust platform for structure-based drug design in NT1 and related hypersomnia disorders.

### 3.9. Treating hyperlipidemia with an orally administered peptide

The determination of the structure of proprotein convertase subtilisin/kexin type 9 (PCSK9) in complex with macrocyclic peptides of the series MK-0616 had critical implications for the design of more effective PCSK9 inhibitors with oral delivery. Enlicitide decanoate, or MK-0616, is a macrocyclic peptide that displays high affinity for PCSK9, thereby preventing its interaction with low-density lipoprotein receptors (LDLR) and promoting the clearance of LDL cholesterol from the bloodstream (Figs. 3 ▸ and 5 ▸*k*; PDB entry 3p5b). Understanding the precise molecular interactions and conformational dynamics of this complex allowed the medicinal chemists to identify key binding sites and features that can be leveraged to optimize the potency and selectivity of new inhibitors (Navia *et al.*, 1989 ▸; Alleyne *et al.*, 2020 ▸; Tucker *et al.*, 2021 ▸). Structural insights gained from the PCSK9–MK-0616 complex series not only guided the design of inhibitors but also underscore the importance of peptide solubility in the overall success of these therapeutic agents in managing hyperlipidemia (Johns *et al.*, 2023 ▸).

Structural biology has impacted from early in discovery with SBDD, to the mid stages of development determining structures of the protein-engineered enzymes to produce the peptide to scale, and late in development with the determination of the structure of the drug substance with macromolecular crystallography and MicroED (Chun *et al.*, 2025 ▸).

### 3.10. Development of monoclonal antibodies and vaccines targeting RSV fusion glycoprotein

The structural studies of the RB1 antibody revealed critical insights into its mechanism of action against respiratory syncytial virus (RSV). X-ray crystallographic analysis of the RB1 Fab fragment complexed with RSV fusion (F) glycoprotein that was engineered to stabilize the pre-fusion conformation demonstrated that the antibody binds at antigenic site IV (Fig. 5 ▸*l*). Notably, the binding mode of RB1 differs substantially from another site IV antibody, 101F, which likely accounts for the superior neutralization potency of RB1, which achieves an IC_50_ of 2.9 ng ml^−1^ against RSV A compared with that of ∼150 ng ml^−1^ for 101F against RSV A. The precise mapping of the epitope of RB1 proved particularly significant given the documented sequence variability in RSV strains over time and the potential for resistance mutations to emerge, a concern underscored by the phase III failure of Suptavumab (REGN2222), a site V-targeting antibody that was discontinued, in part, due to emergence of point mutations in the binding epitope (PDB entry 6ous). These structural insights were instrumental in establishing the clinical viability of RB1 and informing strategies to monitor potential resistance development, ultimately leading to the approval of Enflonsia to prevent RSV in neonates and infants (Tang *et al.*, 2019 ▸).

## 4. Conclusion

### 4.1. Future perspectives: from *ex situ* to *in situ* analysis

The future of structural biology in pharmaceutical development will be shaped by the complementary strengths of X-ray crystallography and SPA cryoEM microscopy. Advances in both fields are enabling increasingly time-resolved and high-resolution visualization of macromolecular dynamics, offering unprecedented insight into how drug targets behave in native or near-native environments. CryoEM continues to expand its impact across structure-based drug discovery as improvements in automation, resolution and AI-assisted interpretation drive higher throughput and broader target coverage. CryoEM is increasingly viewed as an indispensable tool capable of addressing targets that are difficult or impossible to crystallize, while X-ray crystallography remains critical for ultrahigh-resolution structural insights and rigorous validation. Together, these evolving technologies are expected to continue to accelerate drug-discovery pipelines, enhance the precision of ligand design and broaden access to previously intractable therapeutic targets.

The field of structural biology is rapidly transitioning from only *ex situ*, isolated protein studies to an integration with *in situ* investigations that capture macromolecules within their native cellular contexts. In bridging cell biology and structural biology, techniques such as STA Cryo-ET, correlative light and electron microscopy (CLEM), FIB milling and STA now enable the visualization of drug effects, target engagement and pathway modulation directly inside cells and tissues. These approaches complement high-resolution *ex situ* methods (X-ray, single-particle cryo-EM, NMR) by providing spatial context, stoichiometry and mesoscale organization, while advances in sample preparation, labeling and computational reconstruction continue to improve resolution and interpretability. When integrated with chemical biology, proteomics and live-cell imaging, *in situ* structural readouts can connect molecular mechanisms to phenotypic outcomes, closing the loop between target validation, lead optimization and translational relevance.

It is anticipated that the future lies in hybrid workflows that combine *ex situ* precision with *in situ* context, *e.g.* determining high-resolution structures of isolated complexes and then mapping their conformations, interactions and dynamics inside cells via cryo-ET and CLEM. This convergence aims to reduce the gap between biochemical mechanisms and cellular function. The integration and synergy of *ex situ* and *in situ* techniques are poised to elevate the impact of structural biology across the drug discovery and development continuum. By coupling cellular-context structures with high-resolution mechanistic insight and robust computational pipelines, we can move toward mechanism-driven, context-aware therapeutics and more confident, timely decisions. With continued investment in technology, data infrastructure and collaborative practice, real-time, or at least cycle-time-aligned, structural monitoring of therapeutic action becomes an achievable goal, ushering in a new era where structural biology directly guides medicine in its native environment.
